# Case Report: Successful Treatment of Steroid-Refractory Immune Checkpoint Inhibitor-Related Pure Red Cell Aplasia With Cyclosporin

**DOI:** 10.3389/fonc.2020.01760

**Published:** 2020-08-28

**Authors:** Alexandre Gérard, Serena Romani, Elise Van-Obberghen, Audrey Fresse, Marine Muzzone, Nadège Parassol, Annick Boscagli, Fanny Rocher, Delphine Borchiellini, Milou-Daniel Drici

**Affiliations:** ^1^Pharmacovigilance, Department of Clinical Pharmacology, Pasteur Hospital, Centre Hospitalier Universitaire de Nice, Université Côte d’Azur, Nice, France; ^2^Department of Medical Oncology, Centre Antoine Lacassagne, Université Côte d’Azur, Nice, France

**Keywords:** immune checkpoint inhibitor, nivolumab, anemia, pure red cell aplasia, steroid-refractory, cyclosporin, case report

## Abstract

Anemia associated with Immune checkpoint inhibitor (ICI) is usually hemolytic and regenerative. Cases of non-regenerative pure red cell aplasia are rare, and typically improve upon drug discontinuation and after corticotherapy. We herein report a case of nivolumab-related erythroblastopenia refractory to steroids in a melanoma patient that improved only after treatment with cyclosporin. Nivolumab had been well tolerated for 2 months after being introduced as an adjuvant treatment. Hemoglobin level then progressively decreased from 12.7 g/dl as baseline value to a nadir of 4.3 g/dL despite transfusion with a total of 29 packed red blood cells in 3 months. Extensive workup including repeated bone marrow examinations led to the diagnosis of pure red cell aplasia. Anemia persisted despite nivolumab discontinuation and over a month of corticotherapy, but improved dramatically 3 days after cyclosporin initiation and did not recur upon cyclosporin tapering. The patient remains cancer-free 9 months after nivolumab withdrawal. This case highlights the under-recognized risk of erythroblastopenia in patients treated with ICI and proves cyclosporin is a valid alternative for the treatment of steroid-refractory cases.

## Introduction

Nivolumab is an ICI antagonizing the PD-1 that is expressed on the surface of lymphocytes. It is used in the treatment of many cancers, including melanoma (1), as part of adjuvant strategy for high-risk disease or in the advanced or metastatic setting. When bound with its ligand PD-L1, PD1 physiologically downregulates T-cells’ proliferation and cytokines secretion. Since PD-L1 is expressed on many cancer cells, the blockade of PD-1 (mainly expressed on T-cells) by nivolumab enhances the immune system’s ability to recognize and kill malignant cells.

Because of this mechanism of action, most nivolumab-related adverse effects are the result of an excessive immune response against various non-cancerous cells. The most frequently affected organs are skin, digestive tract, liver and endocrine organs (2). Although rare, nivolumab-induced hemolytic anemia has also been described (3), but only few reports concerned a Pure Red Cell Aplasia (PRCA) (4, 5). Early diagnosis of sometimes atypical hematotoxic clinical features (6) is key to an effective management, as ICI-induced hematologic toxicities result in fatal outcomes in 12% of cases (7). Corticosteroids are usually effective against immune-related erythroblastopenia, but second-line treatments of ICI-related adverse events are imperfectly codified in guidelines. We report here the case of a patient treated with adjuvant nivolumab for a melanoma who developed a steroid-refractory PRCA which responded positively to cyclosporin.

## Case Presentation

A 55-year-old woman with a medical history of smoking and peripheral artery disease treated by aspirin was diagnosed with ulcerated acrolentiginous melanoma of the right foot (Breslow 2.1 mm). There was no suspect metabolism highlighted by the PET scan. However, the resected sentinel lymph node proved positive for metastatic cells. Therefore, nivolumab 240 mg every 2 weeks was introduced as adjuvant treatment on May 2019 (day 0).

**FIGURE 1:**
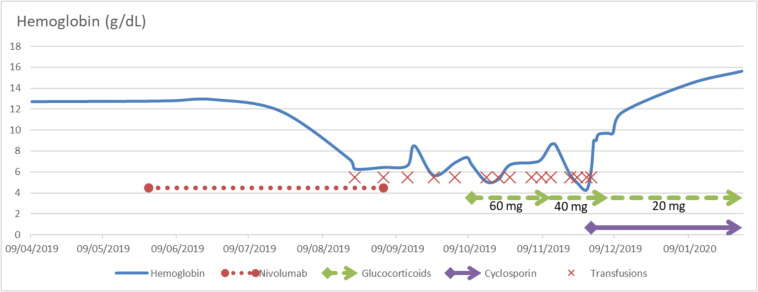
Hemoglobin level as a function of time and treatments. Doses of oral steroids are specified below the respective arrows. Intravenous cyclosporin was introduced at 3 mg/kg for 3 days before being switched for oral cyclosporin 80 mg twice a day with a slow taper.

Before treatment initiation, hemoglobin level was measured at 12.7 g/dL and remained unchanged within the first month of treatment. Two months after nivolumab initiation though, on day 55, hemoglobin level which was 11.8 g/dL, suddenly dropped to 7.2 g/dL on day 84. In the meantime, platelet count dropped to 108 G/L then 93 G/L on day 86 before returning to normal on day 128. Transient and moderate leukopenia at 1.5 G/L also occurred on day 86. The patient was first transfused with 2 packs of red blood cells (PRBCs) followed by transfusions twice a week thereafter. Despite a total of 29 PRBCs with a nadir at 4.3 g/dL and a thorough workup to find a source of bleeding, hemoglobin level remained persistently low (Figure 1).

The patient presented a non-regenerative anemia with a low reticulocytes count. No iron, vitamin B12 or folate deficiency was identified. Haptoglobin level dropped on day 119 and bilirubin level was at some point slightly supranormal. Direct Coombs test turned out to be positive for IgG and C3. Parvovirus B19 serology was found negative. EBV and CMV serologies were consistent with past infections. Hemocultures were negative. There were no paroxysmal nocturnal hemoglobinuria clones. The complement and its fractions were normal. Anti-DNA antibodies were negative. There was no suspect lesion identified at the 18-fluorodeoxyglucose positron emission tomography (FDG-PET) planned exam, but only a diffuse reactional osteomedullary hypermetabolism. Two bone marrow examinations were remarkable for the lack of erythroblastic cell line, without dysplasia or abnormal cells. These findings were consistent with PRCA.

Nivolumab was withdrawn on day 98 after the 8th cycle, and oral corticosteroid treatment was initiated on day 135 (60 mg a day) without efficacy (Figure 1). PRCA persisted and the patient remained transfusions-dependent for almost two more months. On day 184, intravenous cyclosporin was introduced at 3 mg/kg with PRBC transfusion. Cyclosporin was dramatically effective with a hemoglobin level rising to 9 g/dL on the 3rd day and reticulocytes count at 1,246,932/mm^3^. Two weeks after cyclosporin introduction, on day 198, hemoglobin concentration returned to pre-nivolumab levels at 11.7 g/dL. The patient was discharged with oral cyclosporin treatment at 80 mg twice a day (day 278) and corticosteroids both with a slow taper until March 2020. Cyclosporin was first tapered in January (70 mg twice a day) then every 7 to 10 days until March 2020. Corticosteroids were tapered every month between October 2019 and March 2020. On January 2020 (day 226), hemoglobin level was 14.4 g/dL. Nine months after nivolumab withdrawal, there was no PRCA relapse and the patient remained free of melanoma progression without any cancer treatment.

## Discussion

Pure red cell Aplasia is characterized by normocytic normochromic anemia with severe reticulocytopenia and a marked reduction or absence of erythroblasts in otherwise normal bone marrow. Diamond-Blackfan anemia is a genetic congenital PRCA revealed in infancy. Acquired causes of PRCA can be associated with Parvovirus B19 infections, some drugs such as azathioprine, autoimmune diseases, lymphoproliferative disorders and paraneoplastic syndromes (8).

The pathogenesis of acquired PRCA’s is unclear. It may involve the production of antibodies (directed against erythroblasts) (9), T cells or natural killer cells. Patients with acquired PRCA may have increased gamma-delta T cells, and experimental data suggest that those cells inhibit pro-erythroblasts but not common myeloid progenitors (10). In ICI-related cases, activation of cytotoxic T cells is likewise supposed to play a crucial role (5).

When nivolumab is given as monotherapy, anemia has been reported in 5.2% of treated patients without specification of its mechanism (11). There have been several reports of nivolumab-induced regenerative hemolytic anemia, but only few reports of nivolumab-associated PRCA (4, 5). In a descriptive observational study, only one of 35 patients with hematologic immune-related adverse events was diagnosed with PRCA (12). There are 31 cases collated with PD-1 and PD-L1 as a whole (13). Moreover, only 20 cases of nivolumab-induced pure red cell aplasia have been reported in the WHO international safety database “VigiBase^®^”.

In our case, the patient developed PRCA 60 days after nivolumab initiation, which is consistent with time to onset reported in published cases, which ranges from 3 weeks (1 cycle) to 21 months (31 cycles) (5). PRCA can sometimes be explained by an extension of an antibody-mediated immune hemolytic anemia to the bone marrow. Indeed, direct antiglobulin test (Coombs) was positive in our patient, suggesting at first an autoimmune hemolytic anemia. However, haptoglobin level decreased lately and there was no other sign of hemolysis, suggesting a predominantly medullary mechanism, though the contribution of a peripheral mechanism of hemolytic anemia cannot be ruled out. In fact, Coombs positivity may be explained either by autoimmunity activation, antibody-mediated PRCA (14), or a marginal hemolysis contributing to anemia as previously observed (15). Incidentally, osteomedullary hypermetabolism was observed in our patient, reflecting a possible intramedullary inflammatory reaction associated with immune-mediated PRCA.

In published cases, nivolumab-induced anemia was either rapidly fatal or improved after corticotherapy (5). Intravenous immunoglobulin is another treatment of ICI-associated PRCA. It has been successfully used in a case of pembrolizumab-induced PRCA which was cortico-responsive but flared when they were tapered (16) and in a case of corticoresistant ipilimumab-induced PRCA (15). However, in a case of pembolizumab-induced PRCA, oral prednisone and intravenous immunoglobulin poorly improved symptoms (12). An anti-CD52 monoclonal antibody (alemtuzumab) may be another option as it has been more effective than rituximab in treating idiopathic or secondary PRCA, highlighting the importance of T-cells in PRCA pathophysiology (5, 17). Cyclosporin is a potent immunosuppressor known to inhibit T cell-mediated immune reactions, which proved its efficacy in treating other types of acquired PRCA (8, 18). It proved effective in our patient after 3 days only, with a rapid reticulocyte response. This is compatible with a T cell-driven mechanism of nivolumab-generated PRCA.

Nivolumab interruption as well as corticosteroids and cyclosporin did not impair the antitumor response for now. This finding is compatible with suggestions of sustained responses of anti-PD-1 or anti-PD-L1 despite their suspension due to immune-related adverse effects. A meta-analysis shows an association between patient prognosis and the occurrence of immune-related adverse effects (19).

## Conclusion

This case highlights the risk of hypoproliferative anemia in ICI-treated patients, as PRCA had not been observed during the clinical development. A diagnosis of immune PRCA should be suspected when anemia occurs in a patient treated with ICI with low reticulocytes and without obvious vitamin or iron deficiency. If corticosteroids fail to improve PRCA, cyclosporin may be a successful option to consider.

## Data Availability Statement

The raw data supporting the conclusions of this article will be made available by the authors, without undue reservation.

## Ethics Statement

Ethical review and approval was not required for the study on human participants in accordance with the local legislation and institutional requirements. The patients/participants provided their written informed consent to participate in this study. Written informed consent was obtained from the individual(s) for the publication of any potentially identifiable images or data included in this article.

## Author Contributions

All authors contributed to the study conception and design, commented on previous versions of the manuscript, and read and approved the final manuscript. AG, DB, and FR performed the material preparation, data collection, and analysis. AG wrote the first draft of the manuscript.

## Conflict of Interest

The authors declare that the research was conducted in the absence of any commercial or financial relationships that could be construed as a potential conflict of interest.

## Abbreviations

ICI: immune checkpoint inhibitor
PD-1: programmed cell death-1 receptor
Pure Red Cell Aplasia: pure red cell aplasia.
